# Ureteral Rupture Caused by Accidental Intubation of the Ureter with a Foley-Catheter during Ureterorenoscopy

**DOI:** 10.1155/2018/9738265

**Published:** 2018-10-30

**Authors:** F. Löcherbach, P. Grimsehl, A. Sauer, S. Wyler, M. Kwiatkowski

**Affiliations:** Cantonal Hospital Aarau, Aarau, Switzerland

## Abstract

We would like to present the case of a 64-year-old woman who underwent ureterorenoscopy and suffered an iatrogenic ureteral lesion due to an accidental intubation of the left ureter with a Foley-Catheter during the procedure. A Double-J-Stent was implanted into the damaged ureter, and 6 weeks later it fully recovered. To our knowledge there are few similar cases described in the literature with none of those having happened during ureterorenoscopy so far.

## 1. Introduction

Aberrant placement of a Foley-Catheter in the ureter is a very rare but yet described complication of bladder catheterization. There are just 14 [1, 2] cases described in literature, while none of those happened during a ureterorenoscopic procedure. This makes the case at hand a unique one to learn from.

## 2. Case Report

The 64-year-old female patient was initially transferred to our hospital by a smaller district clinic for further treatment after a CT-scan revealed a large left-sided kidney-stone (2 cm) accompanied by an obstructive pyelonephritis. A Double-J-Stent was placed and after successful antibiotic therapy of the pyelonephritis the patient was released from the hospitalization in order to perform a ureterorenoscopic lithotripsy and stone-extraction in an outpatient setting 3 weeks later. Due to the stone's size a complete stone-extraction was not possible in one instance which led us to a second ureterorenoscopy 5 weeks after the initial consultation.

This second procedure started uneventfully with the cystoscopic removal of the Double-J-Stent and the insertion of a guide-wire. Following this the cystoscope was removed in order to insert a standard silicone Ch 12 Foley-Catheter (Nelaton-Tip) blocked with 2.5ml NaCl as it is custom for semirigid ureterorenoscopy in our clinic. Upon entering the bladder with the semirigid instrument, the position of the Foley-Catheter seemed suspicious. Further inspection revealed that the recently placed bladder-catheter led directly into the left-sided ureter. The balloon block was deflated immediately and the catheter was repositioned correctly into the bladder.

**Figure 1:**
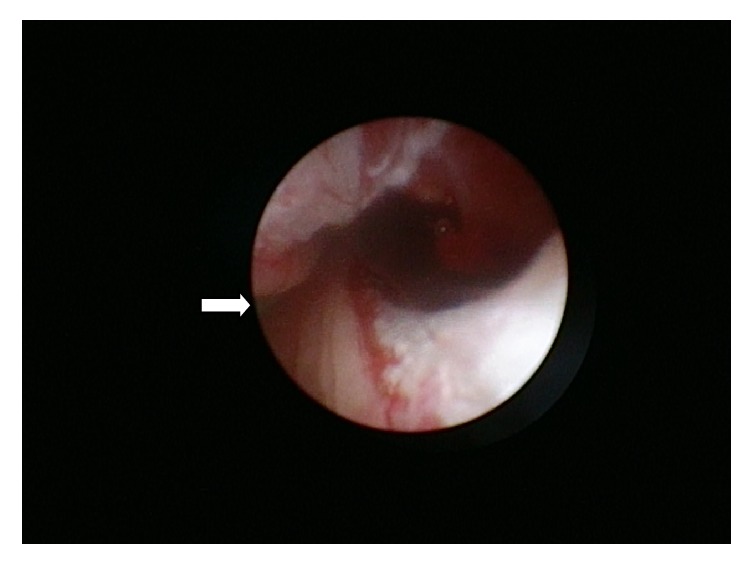
Ureterorenoscopic findings after the misplaced transurethral catheter was removed. The image was taken right after the transurethral catheter's balloon block was deflated and the catheter was pulled back into the bladder. On the left edge of the picture (white arrow) the guide-wire being in situ in the ureter can be seen. In the center of the picture the ruptured ureter shows way into the retroperitoneal space.

**Figure 2:**
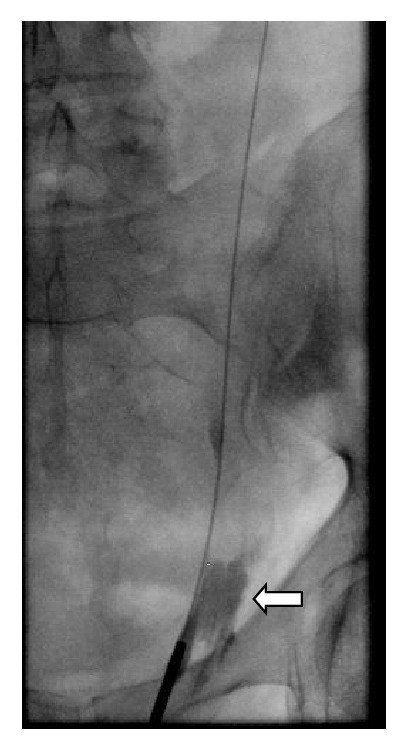
Retrograde contrast application via ureterorenoscope after the misplaced transurethral catheter was removed. The guide-wire previously inserted via Double-J-Stent can be seen in the image's center. After application of contrast there is a leakage (white arrow) into the retroperitoneal space revealing a ruptured ureter.

As demonstrated in Figures 1 and 2, the following ureteroscopy and retrograde ureterography revealed a proximal partial rupture of the left ureter. We therefore decided to reinsert a Double-J-Stent and end the procedure. The already established antibiotic therapy with Ciprofloxacin due to the initial obstructive pyelonephritis preoperatively was continued for another week.

6 weeks later the patient was readmitted for control and in order to complete the stone-extraction. The initially ruptured ureter had recovered completely without contrast-leakage in the retrograde ureterography. The stone-extraction was thus finished without any further intraoperative complications. During the postoperative hospitalization, however, the patient developed a urinary infection which we considered to be caused by the vaporization of the colonized renal stones during the procedure but not directly associated with the initial ureteral injury.

During that hospitalization the Double-J-Stent which was exchanged during the last ureterorenoscopy was also removed. Blood-tests showed unsuspicious renal function and further sonographic controls proved normal renal drainage.

## 3. Discussion

Intraureteral misplacement of a Foley-Catheter is a rare complication of bladder catheterization. There are just 14 cases described in literature so far [1, 2]. It is common sense that women with neurogenic bladder dysfunction and dilated ureteral orifices are at an especially high risk for this complication [2, 3]. According to Ishikawa et al. [2] in 8 of the 14 cases described in literature the patient suffered from a neurogenic bladder dysfunction, 5 of which were female with a total number of 11 female patients. Even without a known neurogenic bladder dysfunction, our patient, being female and having a Double-J-Stent in situ for about 5 weeks before the described event, fits well into this risk profile.

The cases found in literature so far furthermore show that a misplaced Foley-Catheter needs to be diagnosed as fast as possible. Otherwise further complications such as hydronephrosis, pyelonephritis, and consecutive urosepsis can lead to significant long-term damage and even to the patient's death [1, 2, 4, 5].

Concerning further treatment after a misplaced urethral catheter, available data in literature is scarce so far. Most case reports recommend an antibiotic treatment and follow-up CTs as long as there is no rupture of the ureter. In cases of a ruptured ureter on the other hand Double-J-Stenting of the ureter, retrograde or antegrade via nephrostomy, is inevitable. Still we recommend inserting a Double-J-Stent in all cases of intraureteral misplacement of a Foley-Catheter due to the possible posttraumatic swelling of the ureter [1–4, 6].

## 4. Conclusion

Bladder catheterization with a Foley-Catheter is one of the most common procedures in hospitals all around the world. It is a task performed by medical practitioners as well as nurses with a very small amount of complications. Among those complications the accidental intubation of a ureter is one of the most unlikely ones. Nonetheless the case at hand shows that the precise handling of Foley-Catheters as well as a standardized insertion-technique is inevitable to prevent those complications. After the insertion of the catheter one must carefully inflate the balloon and check that this is possible without resistance. Afterwards the catheter can be carefully pulled back onto the bladder-neck. This as well should be possible without a resistance and the catheter should be smoothly moveable within the bladder. If available the correct placement of the catheter should be checked sonographically.

If a Foley-Catheter happens to be misplaced in the ureter, immediate replacement, antibiotic therapy, and Double-J-Stenting of the ureter should be established. The stent itself should be left in situ for at least 4 to 6 weeks. After removal of the Double-J-Stent we recommend a sonographic control or even MRI-/CT-Urography in order to prove an adequate renal drainage. Furthermore close-meshed sonographic follow-ups as well as blood-tests are recommended at least once a year in order to prevent any long-term damage. If the sonographic findings are ambiguous, further investigation by CT-/MRI-Urography or isotope-renogram is advised.
